# Early Patient-Reported Outcomes and Graft-Versus-Host Disease as Indicators of Long-Term Quality of Life and Fatigue After Allogeneic Hematopoietic Stem Cell Transplantation: A 24-Month Prospective Study

**DOI:** 10.3390/cancers18213542

**Published:** 2026-11-04

**Authors:** Gabriela Lampart, Sebastian Klich, Agnieszka Gniadek, Beata Piątkowska-Jakubas

**Affiliations:** 1 Doctoral School of Medical and Health Sciences, Jagiellonian University Medical College, 31-008 Krakow, Poland; 2 Department of Sport Didactics, Wroclaw University of Health and Sport Science, 51-612 Wroclaw, Poland; 3 Institute of Nursing and Midwifery, Faculty of Health Sciences, Jagiellonian University Medical College, 31-008 Krakow, Poland; agnieszka.gniadek@uj.edu.pl; 4 Chair of Hematology, Faculty of Medicine, Jagiellonian University Medical College, 31-008 Krakow, Poland; beata.piatkowska-jakubas@uj.edu.pl

**Keywords:** allogeneic hematopoietic stem cell transplantation, quality of life, fatigue, patient-reported outcomes, graft-versus-host disease, GVHD, survivorship, long-term outcome

## Abstract

Background/Objectives: Long-term recovery after allogeneic hematopoietic stem cell transplantation (allo-HSCT) is heterogeneous, and early patient-reported outcomes (PROs) may provide clinically relevant information about later survivorship. We examined whether quality of life (QoL), fatigue, and early graft-versus-host disease (GVHD) were associated with PROs at 24 months after allo-HSCT. Methods: Adult allo-HSCT recipients were prospectively followed at six predefined timepoints up to 24 months. The primary early landmark was 6 months (T3), and the primary long-term outcome was 24-month (T6) QoL assessed using the Functional Assessment of Cancer Therapy—Bone Marrow Transplant (FACT-BMT). Fatigue was assessed using the Brief Fatigue Inventory (BFI) and Functional Assessment of Chronic Illness Therapy—Fatigue (FACIT-F). Associations were examined using parsimonious regression models including early GVHD. Results: Twenty-eight participants had PRO data at both T3 and T6. Higher FACT-BMT at T3 was associated with higher FACT-BMT at T6 after adjustment for early GVHD (B = 1.077, 95% CI 1.019–1.135, *p* < 0.001). Early GVHD remained associated with lower T6 FACT-BMT (B = −6.133, 95% CI −9.373 to −2.893, *p* < 0.001). Higher BFI and lower FACIT-F at T3 were associated with poorer T6 QoL, while 6-month fatigue was also associated with corresponding 24-month fatigue outcomes. Among patients with GVHD, non-responders had less favorable 24-month fatigue outcomes than responders. Conclusions: PROs at 6 months were strongly associated with 24-month QoL and fatigue. The 6-month assessment may represent a clinically informative survivorship landmark, although larger multicenter studies are needed to confirm these associations.

## Simple Summary

Long-term recovery after allogeneic hematopoietic stem cell transplantation varies substantially between patients. In this prospective study, we examined whether patient-reported quality of life and fatigue assessed six months after transplantation were associated with outcomes two years later. Better quality of life at six months was strongly associated with better quality of life at 24 months, while greater fatigue at six months was associated with poorer long-term quality of life and greater fatigue. Early graft-versus-host disease was also associated with less favorable long-term patient-reported outcomes. Among patients who developed graft-versus-host disease, those who did not respond to treatment showed poorer fatigue-related outcomes at 24 months than responders. These findings suggest that patient-reported outcomes assessed during early recovery may provide clinically useful information about longer-term survivorship and could help identify patients who may benefit from closer follow-up and supportive care.

## 1. Introduction

Allogeneic hematopoietic stem cell transplantation (allo-HSCT) offers the possibility of long-term disease control or cure for patients with hematological malignancies and other severe hematological disorders. However, survivorship after transplantation is characterized by substantial heterogeneity in physical functioning, symptom burden, and quality of life (QoL). Although many patients experience gradual improvement after the intensive early post-transplant period, recovery is not uniform, and a proportion of survivors continue to report persistent fatigue, functional limitations, and impaired QoL months or years after transplantation [1,2,3,4]. Recent long-term survivorship data further demonstrate considerable variability in patient-reported health after allo-HCT, underscoring the need to identify individuals at risk of less favorable long-term recovery [4].

Patient-reported outcomes (PROs) may provide clinically relevant information that is not fully captured by conventional medical variables alone. Previous studies have reported associations between pre-transplant or early post-transplant PROs and subsequent clinical outcomes after hematopoietic cell transplantation. Johnson et al. found that poorer baseline quality of life and greater fatigue were associated with clinically relevant post-transplant complications, including acute graft-versus-host disease (GVHD) [5]. Kurosawa et al. similarly demonstrated that pre-transplant QoL was associated with subsequent outcomes and that individual QoL domains followed distinct patterns during two years of prospective follow-up [6]. However, evidence regarding the independent prognostic contribution of PROs is not uniform. In an exploratory analysis of 117 allo-HSCT recipients, Gudmundstuen et al. found that baseline PROs generally did not retain independent associations with one-year overall survival or non-relapse mortality after adjustment for established clinical and sociodemographic factors [7].

Fatigue may be particularly relevant in this context. It is among the most prevalent and persistent symptoms following allo-HSCT and is closely associated with impaired physical and functional well-being [8,9]. In a prospective study with up to six years of follow-up, greater fatigue interference assessed approximately 100 days after allogeneic HCT was independently associated with increased mortality risk, suggesting that early symptom burden may contain information extending beyond the immediate post-transplant period [8]. Longitudinal studies have also demonstrated that fatigue may remain elevated relative to population norms for several years after transplantation and may be influenced by transplant-related complications, including chronic GVHD [9]. More recent survivorship data similarly indicate that fatigue can remain an important long-term burden among allo-HCT survivors. Together, these observations support examining fatigue not only as an outcome of transplantation but also as a potential early indicator of subsequent patient-reported health [4].

GVHD is another major factor shaping long-term recovery after allo-HSCT. Chronic GVHD remains an important source of late morbidity and is associated with substantial impairment in functional status and quality of life [10,11]. Greater chronic GVHD severity has been associated with poorer QoL across multiple domains, particularly physical functioning [10], while survivors with active chronic GVHD have reported worse QoL than those with resolved disease or no history of chronic GVHD [12]. Patient and clinician assessments may also provide partly distinct information. In a large chronic GVHD cohort, patient-reported treatment response at six months showed only limited agreement with clinician- and NIH-defined response but was associated with subsequent failure-free survival [13]. Recent reviews likewise emphasize that PROs can capture symptom burden and functional consequences of chronic GVHD that may not be fully reflected by clinician-rated disease measures [14].

Despite growing interest in PROs after allo-HSCT, much of the existing prognostic literature has focused on pre-transplant assessments or on subsequent clinical outcomes such as mortality, non-relapse mortality, relapse, or transplant-related complications [5,6,7,8,15]. Longitudinal studies have described changes in QoL and symptom burden during the months and years following transplantation [2,3,4,6,9,15], but a different clinical question remains less well characterized: whether patient-reported status assessed after the most intensive early recovery period is prospectively associated with patient-reported health later in survivorship. In particular, comparatively little is known about whether QoL and fatigue at approximately six months after allo-HSCT are associated with QoL and fatigue at 24 months. The six-month assessment represents a clinically relevant landmark because patients have progressed beyond the most intensive early post-transplant period while remaining within a timeframe in which persistent symptoms and functional limitations may warrant continued supportive care. Contemporary expert recommendations also support longitudinal PRO assessment after HCT, including assessment around day 180 and during subsequent survivorship follow-up [16].

Therefore, the present prospective study aimed to examine whether patient-reported QoL and fatigue assessed during the early post-transplant period, with particular emphasis on the six-month assessment, were associated with QoL and fatigue 24 months after allo-HSCT. We additionally examined whether early GVHD characteristics, including response to GVHD treatment where applicable, were associated with long-term patient-reported outcomes. We hypothesized that lower QoL at six months would be associated with lower QoL at 24 months. We further hypothesized that greater fatigue at six months and an unfavorable early GVHD course would be associated with poorer patient-reported outcomes at 24 months. Secondary exploratory analyses evaluated whether patient-reported status immediately after transplantation and the magnitude of change during the first six months provided additional information regarding long-term outcomes.

## 2. Materials and Methods

### 2.1. Study Design

This prospective observational study was conducted at the Department of Hematology, University Hospital in Krakow, Poland, as part of a larger longitudinal project evaluating patient-reported outcomes (PROs) after allogeneic hematopoietic stem cell transplantation (allo-HSCT). The parent project included six predefined assessment points extending from the immediate post-transplant period to 24 months after transplantation. Adult patients aged ≥18 years undergoing allo-HSCT were consecutively recruited prior to transplantation and provided written informed consent.

The present analysis was designed to investigate prospective associations between early patient-reported status and outcomes at 24 months after allo-HSCT. Unlike the early-phase analysis of the parent cohort, which focused on PRO trajectories during the first six months after transplantation, the current study used the six-month assessment as the primary early landmark and the 24-month assessment as the principal long-term outcome timepoint. Assessments at 12 and 18 months formed part of the parent longitudinal protocol but were not included in the primary analyses of the present manuscript, which specifically addressed the relationship between early status and 24-month outcomes.

The present study represents a secondary analysis of a prospectively followed cohort, and the available sample size was determined by the number of eligible participants enrolled in the parent longitudinal study and by availability of 24-month follow-up data. No separate a priori sample-size calculation was performed specifically for the present early-to-long-term association analysis.

Given the expected limited number of participants with complete long-term data, multivariable models were deliberately parsimonious. The primary models were restricted to the principal early PRO indicator and early GVHD status. Additional clinical variables were introduced only in separate exploratory or sensitivity models rather than simultaneously in a single highly parameterized model. This approach was selected to reduce the risk of overfitting and unstable regression estimates.

The study was conducted in accordance with the Declaration of Helsinki and was approved by the Bioethics Committee of the Jagiellonian University Medical College (approval no. 1072.6120.75.2022). All participants provided written informed consent before study participation. The study was observational and did not introduce interventions beyond routine clinical care.

Early clinical variables previously characterized in the cohort were examined as additional exploratory indicators of 24-month outcomes. Particular attention was given to duration of hospitalization and documented weight loss. Age was categorized according to the cohort mean as younger (<48 years) and older (≥48 years), while duration of hospitalization was categorized as shorter (<42 days) and longer (≥42 days), using the cohort mean of 42 days as the cut-off. These categorizations were consistent with the previous analysis of the same cohort. Age group was used for descriptive characterization of the analytical cohort rather than as a covariate in the primary regression models.

Because of the limited cohort size, hospitalization duration and documented weight loss were not entered simultaneously with multiple additional covariates in the primary models. Instead, their contribution was examined in separate parsimonious sensitivity models by adding each factor individually to the primary model: FACT-BMT T6 = FACT-BMT T3 + early GVHD + hospitalization group and FACT-BMT T6 = FACT-BMT T3 + early GVHD + documented weight loss.

These models were considered exploratory and were used to assess whether the association between early PRO status and the 24-month outcome remained broadly consistent after consideration of selected clinical characteristics. No automated or stepwise variable-selection procedures were used.

### 2.2. Participants

Participants were eligible for a given primary analysis when both the relevant early indicator and corresponding 24-month outcome were available. The number of participants contributing to each analysis was therefore determined separately according to data availability. Reasons for absence of 24-month data, including death, loss to follow-up, change of treatment center, or lack of further contact, were recorded separately whenever available. The final primary analytical cohort consisted of 28 patients.

### 2.3. Assessment Schedule

PROs were collected at six predefined timepoints: within 24 h after allo-HSCT (T1), at 1 month (T2), 6 months (T3), 12 months (T4), 18 months (T5), and 24 months (T6). Assessments were performed during hospitalization or routine outpatient follow-up visits, and questionnaires were completed independently by participants, with clarification provided by research staff when necessary. The T1 assessment was performed after conditioning and transplantation and was therefore considered an immediate post-transplant assessment rather than a true pre-transplant baseline.

For the present study, T3 at 6 months was prespecified as the primary early landmark, because it represents a clinically relevant stage after the most intensive early post-transplant period while remaining sufficiently early to characterize persistent patient-reported burden. T6 at 24 months was prespecified as the primary long-term outcome assessment. T1 measurements and changes between T1 and T3 were reserved for secondary exploratory analyses. T2 was not included in the analyses of the present study.

### 2.4. Patient-Reported Outcome Measures

Quality of life was assessed using the Functional Assessment of Cancer Therapy—Bone Marrow Transplant (FACT-BMT). The instrument incorporates the FACT—General (FACT-G) domains of physical well-being, social/family well-being, emotional well-being, and functional well-being together with bone marrow transplantation-specific concerns. Higher FACT-BMT scores indicate better patient-reported quality of life. The same validated instrument was used consistently throughout the longitudinal project.

Fatigue was evaluated using two complementary validated instruments. The Functional Assessment of Chronic Illness Therapy—Fatigue (FACIT-F) is a 13-item measure in which higher scores indicate lower levels of fatigue. The Brief Fatigue Inventory (BFI) assesses fatigue severity and interference with daily functioning, with higher scores indicating greater fatigue. Both instruments were administered at the predefined longitudinal assessment points. The Brief Fatigue Inventory (BFI) global score was calculated as the arithmetic mean of nine items rated on an 11-point numerical scale (0–10), including three items assessing fatigue severity and six items evaluating fatigue-related interference with daily functioning. The initial yes/no screening question was excluded from the calculation. Higher scores indicate greater fatigue severity and interference. In the early-GVHD non-responder subgroup, all six recorded BFI scores at T3 were 7.0; therefore, the group standard deviation was 0.00. No imputation was applied to these observed values.

### 2.5. Early GVHD and Treatment-Response Assessment

Clinical information on graft-versus-host disease (GVHD) was obtained from medical records. GVHD was assessed during post-transplant hospitalization and routine follow-up and was classified according to the clinical documentation, taking into account timing, clinical presentation, and documented disease characteristics. GVHD assessment and evaluation of treatment response were supported by the eGVHD application (UZ Leuven, Belgium), used in accordance with clinical recommendations. The source definition was kept consistent with the earlier analysis from the same cohort.

For the present study, early GVHD was defined as acute or chronic GVHD occurring during the early follow-up period between T1 and T3. Because this classification was established during follow-up, early GVHD should be interpreted as a follow-up-defined clinical characteristic rather than as an exposure already present at T1. The term “early GVHD” represents a composite history of documented acute and/or chronic GVHD during the T1–T3 observation period. It does not represent active GVHD at 24 months. Because the numbers of patients with each GVHD phenotype were small, the pooled early-GVHD coefficient was interpreted as an association with a history of early GVHD, not as a type-specific or causal effect.

Where applicable, GVHD type, predominant organ involvement, severity, and treatment response were extracted from the medical documentation. Treatment response was defined according to the best documented response by the end of the early follow-up period. Complete response and partial response were combined and classified as response, whereas no response, disease progression, or lack of documented clinical improvement were classified as non-response. This definition was retained from the previous early-phase analysis to ensure consistency across reports from the same longitudinal project.

Treatment-response analyses were prespecified as secondary or exploratory because the number of patients with GVHD, and particularly the number of responders and non-responders with available T6 outcomes, was expected to be limited. These analyses were interpreted as associations between clinical subgroups and long-term outcomes and not as causal estimates of the effect of GVHD treatment.

Among participants who developed GVHD during early follow-up, treatment response was examined as an additional clinical factor. Complete and partial responses were combined as a responder category, whereas absence of response, progression, or absence of documented improvement constituted the non-responder category, consistent with the clinical classification used for the same early GVHD episodes in the previous analysis of this cohort.

Because of the expected small size of the GVHD subgroup, treatment-response analyses were considered secondary or exploratory. Twenty-four-month FACT-BMT, BFI, and FACIT-F scores were summarized separately for responders and non-responders. Where subgroup size and distribution of the outcome permitted, unadjusted regression models or between-group estimates with 95% CIs were used to quantify differences. Multivariable treatment-response models were not fitted if subgroup numbers were considered insufficient to provide stable estimates.

### 2.6. Primary and Secondary Outcomes

The primary outcome was FACT-BMT total score at 24 months after allo-HSCT (T6). The key secondary outcomes were BFI and FACIT-F scores at T6. The analysis focused on continuous PRO scores. Individual participants were not categorized as improved, stable, or deteriorated according to minimum clinically important difference thresholds, and no individual recovery phenotypes were derived, as these approaches were outside the scope of the present study.

The primary analysis examined the association between FACT-BMT at 6 months (T3) and FACT-BMT at 24 months (T6). The unadjusted relationship was initially characterized using scatterplots, correlation coefficients, and simple linear regression. The primary multivariable model was specified as: FACT-BMT T6 = FACT-BMT T3 + early GVHD status where FACT-BMT T6 was the dependent variable and FACT-BMT T3 and early GVHD status were the prespecified explanatory variables. Early GVHD status was coded according to the occurrence of GVHD during the predefined early post-transplant follow-up period, using the same underlying clinical classification as in the earlier analysis of the cohort.

The regression coefficient for FACT-BMT T3 was interpreted as the expected difference in 24-month FACT-BMT associated with a one-unit difference in the 6-month score, conditional on early GVHD status. Unstandardized regression coefficients (B), 95% CIs, and *p*-values were reported. Standardized regression coefficients (β) were additionally considered to facilitate comparison of the relative magnitude of associations across PRO measures.

To evaluate whether early fatigue was associated with subsequent QoL, two additional prespecified models were fitted: FACT-BMT T6 = BFI T3 + early GVHD status and FACT-BMT T6 = FACIT-F T3 + early GVHD status.

BFI and FACIT-F were analyzed in separate models because the two measures assess closely related aspects of fatigue and were expected to be strongly correlated. They were therefore not entered simultaneously into the same multivariable model.

These analyses were considered key secondary analyses and were intended to determine whether fatigue burden at approximately 6 months after allo-HSCT was associated with patient-reported QoL 18 months later.

Secondary analyses examined whether fatigue assessed at T3 was associated with the corresponding fatigue outcome at T6. Separate models were fitted for the two fatigue instruments: BFI T6 = BFI T3 + early GVHD status and FACIT-F T6 = FACIT-F T3 + early GVHD status.

This approach allowed persistence of fatigue burden between 6 and 24 months to be assessed while accounting for the presence of early GVHD. Cross-instrument combinations were not included in the same model to avoid unnecessary model complexity and collinearity.

### 2.7. Candidate Early Indicators

The primary early indicator was FACT-BMT total score at T3 (6 months). Key secondary early indicators were BFI and FACIT-F scores at T3 and early GVHD status.

The primary prospective association of interest was therefore: FACT-BMT at 6 months → FACT-BMT at 24 months.

Secondary analyses evaluated whether fatigue at 6 months was associated with 24-month QoL and corresponding 24-month fatigue outcomes. Early GVHD was evaluated as a complementary clinical factor. Where sample size permitted, early GVHD treatment response was examined as an additional clinical indicator.

PROs assessed immediately after transplantation at T1 and the magnitude of change between T1 and T3 were prespecified as exploratory indicators. These analyses included changes in FACT-BMT, BFI, and FACIT-F during the first six months. Because T1 was not a true pre-transplant baseline, these analyses were interpreted specifically as associations involving the immediate post-transplant state or early post-transplant change.

### 2.8. Missing Data, Attrition and Exploratory Analyses

The present analysis focused on the prospective association between early post-transplant patient-reported outcomes and outcomes assessed 24 months after allo-HSCT. The 6-month assessment (T3) was defined a priori as the primary early landmark, whereas the 24-month assessment (T6) represented the long-term outcome timepoint. The 12- and 18-month assessments (T4 and T5) were not included in the primary analyses because the objective of the present study was to evaluate early-to-long-term associations rather than longitudinal late-phase trajectories.

For each analysis, participants were included if the relevant early indicator and corresponding 24-month outcome were available. No imputation of missing patient-reported outcome data was performed. Participants who died before T6 were therefore not assigned hypothetical 24-month PRO values.

Participant flow from the parent cohort to the 24-month analytical sample was documented, including deaths, loss to follow-up, change of treatment center or lack of further contact, and other reasons for unavailable long-term assessments. To explore the potential direction of attrition and survivor bias, participants with available long-term PRO data were descriptively compared with participants without complete follow-up using available early demographic, clinical, and PRO information. This comparison was intended to characterize potential selection effects rather than to test formal hypotheses.

The primary analyses were therefore interpreted as describing prospective associations among participants with available 24-month patient-reported outcomes rather than as representing the complete original allo-HSCT cohort.

Although T3 was the prespecified primary landmark, exploratory analyses examined whether PRO status immediately after transplantation (T1) was associated with outcomes at T6. T1 was interpreted as an immediate post-transplant assessment rather than as a true pre-transplant baseline because it was obtained within 24 h after allo-HSCT and after completion of conditioning.

Exploratory analyses additionally examined the magnitude of change between T1 and T3. Change scores were calculated as: ΔPRO = PRO T3 − PRO T1.

The direction of change was interpreted according to the scoring properties of each instrument: positive change indicated improvement for FACT-BMT and FACIT-F, whereas a decrease in BFI represented improvement in fatigue.

Associations between early change and T6 outcomes were treated as exploratory because change scores may be influenced by baseline values, measurement variability, and regression to the mean. These analyses were not used to define individual recovery categories or clinically meaningful recovery phenotypes.

### 2.9. Statistical Analysis

All statistical analyses were performed using IBM SPSS 26 Statistics (IBM Corp., Armonk, NY, USA). Statistical tests were two-sided, and a *p*-value < 0.05 was considered statistically significant for the primary analysis. Because secondary and exploratory analyses were prespecified to provide supportive rather than confirmatory evidence, no formal adjustment for multiple comparisons was applied; these findings were interpreted primarily on the basis of effect estimates, 95% confidence intervals (CIs), and consistency across outcomes.

Continuous variables were summarized using means and standard deviations (SDs) and, where distributions showed substantial asymmetry or outlying observations, additionally using medians and interquartile ranges (IQRs). Categorical variables were presented as frequencies and percentages. Patient-reported outcomes were analyzed using their original continuous scoring scales. Higher FACT-BMT and FACIT-F scores represented better quality of life and lower fatigue burden, respectively, whereas higher BFI scores indicated greater fatigue.

Linear regression assumptions were evaluated using graphical and numerical diagnostics. Linearity was assessed through scatterplots and residual plots, and the distribution of model residuals was examined using histograms and normal Q–Q plots.

Residual normality and heteroscedasticity were additionally assessed using the Shapiro–Wilk and Breusch–Pagan tests, respectively. Heteroscedasticity-consistent HC3 standard errors were examined as a robustness analysis for the prespecified regression models.

Potentially influential observations were evaluated using standardized or studentized residuals, leverage values, and Cook’s distance. Observations were not excluded solely on the basis of statistical influence measures. Where a potentially influential observation materially affected a model estimate, sensitivity analyses with and without that observation were considered and any relevant differences were reported.

Multicollinearity was evaluated using tolerance and variance inflation factors (VIFs). Variables showing substantial conceptual overlap or high collinearity were not included simultaneously. In particular, BFI and FACIT-F were modeled separately.

Given the limited sample size, no interaction terms were included in the primary multivariable models. In particular, PRO × GVHD interactions were not routinely tested because such models would require substantially larger subgroup sizes for stable estimation.

For regression models, unstandardized regression coefficients (B), 95% CIs, and exact *p*-values were reported. Where appropriate, standardized regression coefficients (β) and model R^2^ were additionally presented. For group-based analyses, mean differences and corresponding 95% CIs were prioritized over *p*-values alone.

The present analyses were designed to evaluate prospective associations and early indicators of long-term patient-reported health. They were not intended to develop, internally validate, or externally validate a clinical prediction model, and measures of predictive performance such as ROC curves, classification accuracy, or risk scores were therefore not used.

To assess the stability of the reported associations, the five prespecified two-predictor linear regression models were independently re-estimated from participant-level data in Python 3. Leave-one-out cross-validation (LOOCV) was performed by repeatedly fitting each model in 27 participants and evaluating the prediction for the excluded participant. The cross-validated coefficient of determination (Q^2^) was calculated as 1 − Σ(y_i_ − ŷ_−i_)^2^/Σ(y_i_ − ȳ)^2^, and root mean squared error (RMSE) was also reported. Participant-level nonparametric bootstrap resampling (2000 replicates) was used to estimate percentile 95% confidence intervals for regression coefficients. Variance inflation factors were calculated for each predictor. Potentially influential cases were flagged using Cook’s distance > 4/n, with refitting after their omission performed only as a sensitivity analysis. These procedures were used as internal stability checks and were not considered external validation of a clinical prediction model.

## 3. Results

### 3.1. Participant Flow and Characteristics of the Analytical Cohort

**Figure 1:**
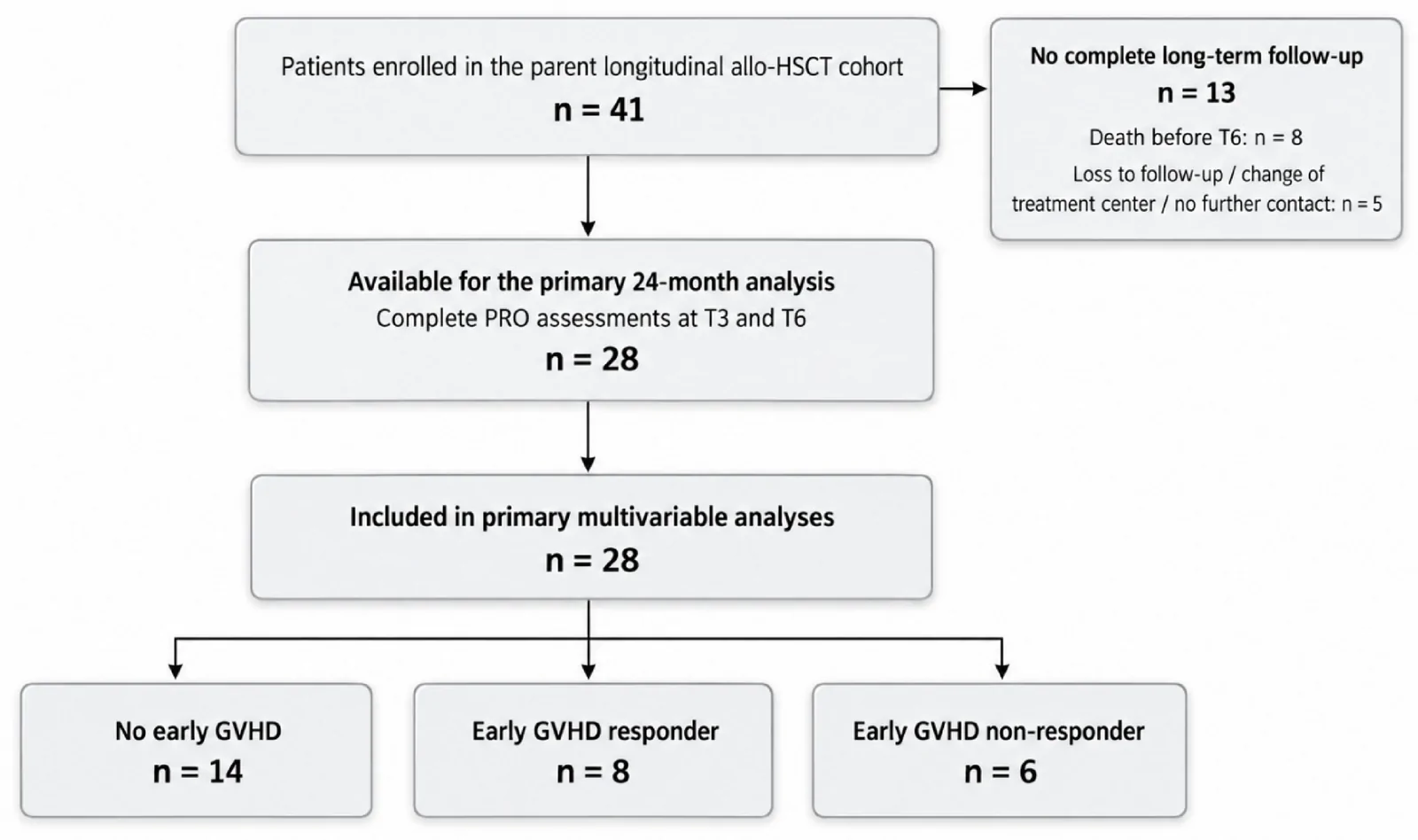
Participant flow from the parent longitudinal cohort to the 24-month analytical sample and early GVHD response subgroups.

A total of 41 patients were enrolled in the parent longitudinal allo-HSCT project. Twenty-eight participants had available FACT-BMT data at both the 6-month (T3) and 24-month (T6) assessments and were included in the primary analytical cohort. Of the 13 participants without complete long-term follow-up, eight died before completion of follow-up and five were lost to follow-up, changed treatment center, or had no further contact (Figure 1).

The analytical cohort comprised 17 women (60.7%) and 11 men (39.3%). Thirteen participants (46.4%) belonged to the younger age group and 15 (53.6%) to the older age group. The most frequent diagnosis was acute myeloid leukemia (n = 16, 57.1%), followed by chronic myeloid leukemia (n = 7, 25.0%), acute lymphoblastic leukemia (n = 4, 14.3%), and myelodysplastic syndrome (n = 1, 3.6%).

Early GVHD occurred in 14 participants (50.0%). Among patients who developed GVHD, eight were classified as responders to treatment and six as non-responders. Documented weight loss was recorded in 15 participants (53.6%), whereas 12 participants (42.9%) were classified as having a longer hospitalization. Participant characteristics are summarized in Table 1.

### 3.2. Patient-Reported Outcomes at 6 and 24 Months

Overall QoL was higher at the 24-month assessment than at the 6-month assessment. Mean FACT-BMT was 104.11 ± 28.47 at T3 and 114.18 ± 33.05 at T6. Mean BFI was 5.50 ± 1.20 at T3 and 5.20 ± 1.57 at T6, whereas mean FACIT-F was 24.79 ± 7.45 and 27.91 ± 9.28, respectively.

Differences were observed according to early GVHD status and treatment response. Among patients without early GVHD, mean FACT-BMT was 123.57 ± 19.33 at T3 and 138.21 ± 19.87 at T6. Corresponding values were 92.38 ± 20.72 and 100.38 ± 22.66 among GVHD responders and 74.33 ± 21.48 and 76.50 ± 22.95 among GVHD non-responders.

A similar pattern was observed for fatigue. Mean BFI values at T3/T6 were 4.71 ± 0.83/4.08 ± 0.93 in patients without GVHD, 5.75 ± 1.04/5.66 ± 1.26 in GVHD responders, and 7.00 ± 0.00/7.22 ± 0.23 in non-responders. Mean FACIT-F values were 30.43 ± 4.31/35.21 ± 4.92, 21.25 ± 5.85/23.62 ± 6.31, and 16.33 ± 2.94/16.58 ± 3.65, respectively (Table 2). At T3, the recorded BFI score was 7.0 for each of the six participants classified as GVHD non-responders, yielding a within-group standard deviation of 0.00.

### 3.3. Association Between 6-Month QoL and 24-Month QoL

FACT-BMT assessed at 6 months was strongly positively correlated with FACT-BMT at 24 months (r = 0.994, *p* < 0.001). In univariable linear regression, each one-point higher FACT-BMT score at T3 was associated with a 1.154-point higher FACT-BMT score at T6 (B = 1.154, 95% CI 1.102–1.205, *p* < 0.001), with the model explaining 98.8% of the variance in the 24-month outcome. Univariable associations between 6-month PROs and 24-month FACT-BMT are summarized in Table 3.

In the prespecified primary multivariable model including FACT-BMT at T3 and early GVHD status, the association between 6-month and 24-month QoL remained strong. Each one-point higher FACT-BMT score at T3 was associated with a 1.077-point higher FACT-BMT score at T6 (B = 1.077, 95% CI 1.019–1.135, *p* < 0.001; β = 0.928).

**Figure 2:**
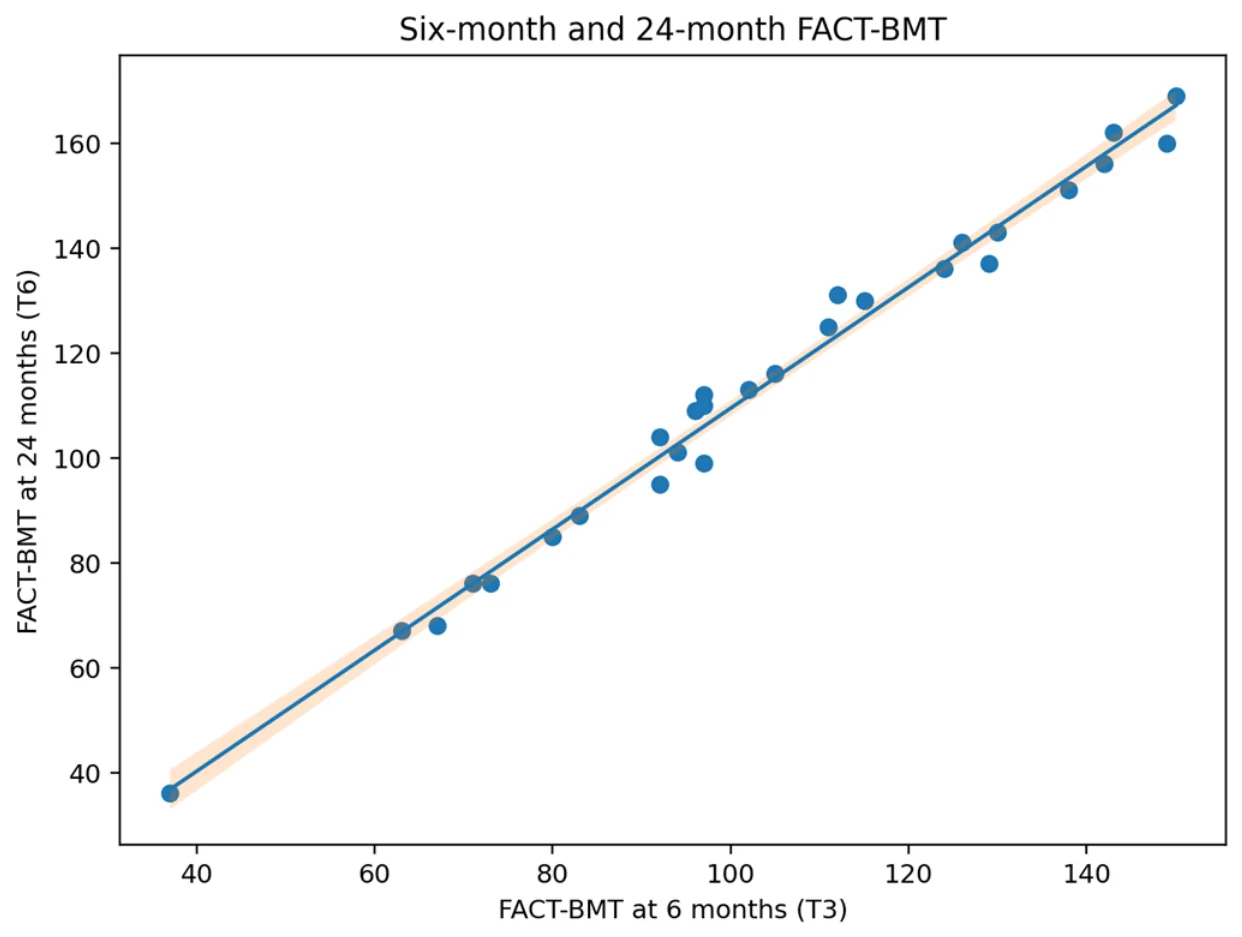
Association between FACT-BMT at six months (T3) and 24 months (T6). Each point represents one participant (n = 28). The solid line shows the fitted univariable ordinary least-squares regression, and the shaded area represents the 95% confidence interval for the mean fitted response.

Early GVHD remained associated with lower 24-month QoL after adjustment for 6-month FACT-BMT. After adjustment for FACT-BMT at T3, patients who had developed early GVHD had a mean FACT-BMT score at T6 that was 6.13 points lower than that of patients without early GVHD (B = −6.133, 95% CI −9.373 to −2.893, *p* < 0.001). The primary model explained 99.2% of the variance in FACT-BMT at T6 (R-squared = 0.992). The adjusted model estimates are presented in Table 4, and the relationship between FACT-BMT at T3 and T6 is illustrated in Figure 2. Individual paired FACT-BMT scores at T3 and T6, stratified by early GVHD status, are additionally presented in Supplementary Figure S1. In the independent participant-level reanalysis, the primary-model in-sample R^2^ was 0.9924. In LOOCV, the model achieved Q^2^ = 0.9905 and an RMSE of 3.16 FACT-BMT points. The coefficient for FACT-BMT at T3 remained positive in 2000 bootstrap samples, with a percentile 95% interval of 1.014–1.127. These internal stability analyses are summarized in Supplementary Table S1 and should not be interpreted as establishing external predictive performance.

### 3.4. Early Fatigue and Long-Term QoL

Greater fatigue at 6 months was associated with poorer QoL at 24 months. BFI at T3 showed a strong inverse correlation with FACT-BMT at T6 (r = −0.798, *p* < 0.001). In univariable analysis, each one-point higher BFI score at T3 was associated with a 21.94-point lower FACT-BMT score at T6 (B = −21.936, 95% CI −28.622 to −15.250, *p* < 0.001).

After adjustment for early GVHD, BFI at T3 remained associated with FACT-BMT at T6 (B = −15.046, 95% CI −23.149 to −6.943, *p* < 0.001; β = −0.547). Early GVHD also remained associated with lower FACT-BMT in this model after adjustment for BFI at T3 (B = −24.428, 95% CI −43.553 to −5.302, *p* = 0.014). The model explained 71.5% of the variance in 24-month FACT-BMT (R-squared = 0.715).

FACIT-F at T3 was positively correlated with FACT-BMT at T6 (r = 0.870, *p* < 0.001). In univariable analysis, each one-point higher FACIT-F score at T3 was associated with a 3.861-point higher FACT-BMT score at T6 (B = 3.861, 95% CI 2.980–4.742, *p* < 0.001).

**Figure 3:**
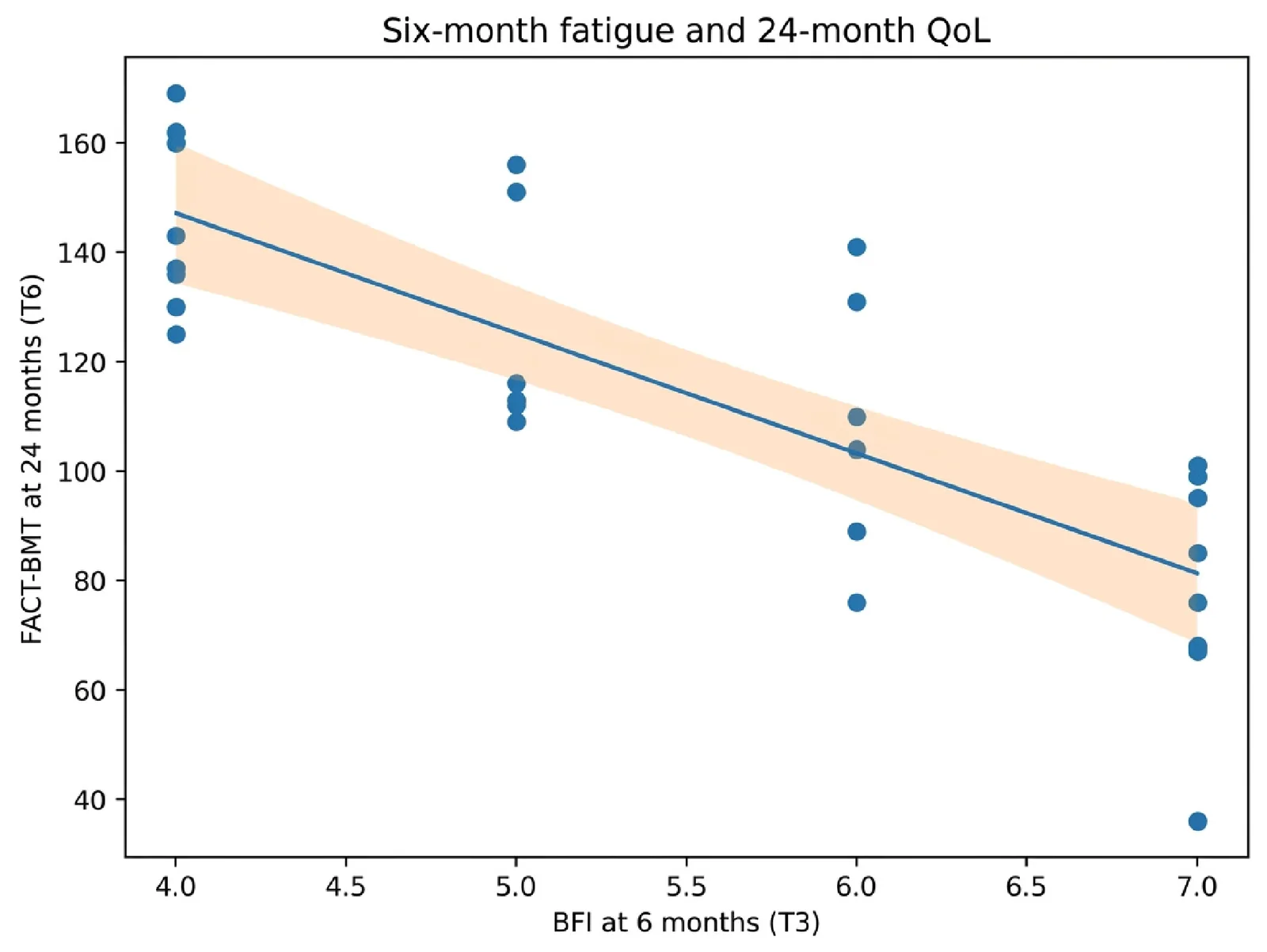
Association between Brief Fatigue Inventory (BFI) at six months (T3) and FACT-BMT at 24 months (T6). Each point represents one participant (n = 28). The solid line represents the fitted univariable linear regression, and the shaded area the 95% confidence interval for the mean fitted response. Higher BFI indicates greater fatigue.

**Figure 4:**
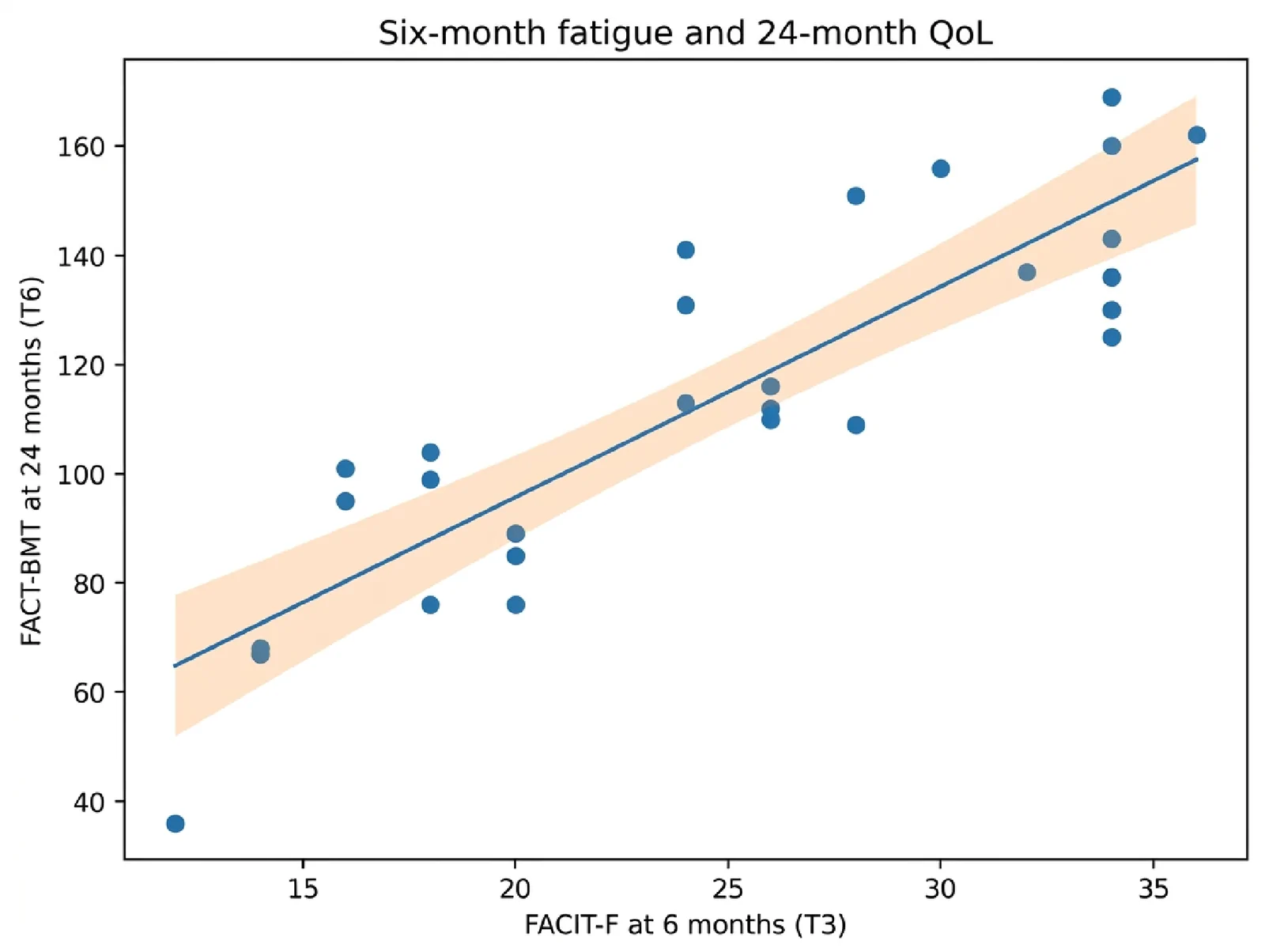
Association between Functional Assessment of Chronic Illness Therapy—Fatigue (FACIT-F) at six months (T3) and FACT-BMT at 24 months (T6). Each point represents one participant (n = 28). The solid line represents the fitted univariable linear regression, and the shaded area the 95% confidence interval for the mean fitted response. Higher FACIT-F indicates lower fatigue.

After adjustment for early GVHD, FACIT-F at T3 remained strongly associated with FACT-BMT at T6 (B = 3.275, 95% CI 1.896–4.654, *p* < 0.001; β = 0.738). In contrast, early GVHD was not associated with FACT-BMT after adjustment for FACIT-F at T3 (B = −11.110, 95% CI −31.289 to 9.069, *p* = 0.268). This model explained 76.9% of the variance in FACT-BMT at T6 (R-squared = 0.769). The corresponding adjusted model estimates are presented in Table 4, and these relationships are illustrated in Figure 3 and Figure 4.

### 3.5. Persistence of Fatigue from 6 to 24 Months

Fatigue at 6 months was strongly associated with fatigue at 24 months. In the model with BFI at T6 as the dependent variable, BFI at T3 remained associated with higher BFI at T6 after adjustment for early GVHD (B = 1.142, 95% CI 1.000–1.284, *p* < 0.001; β = 0.876). Early GVHD was additionally associated with a 0.46-point higher BFI score at T6 (B = 0.455, 95% CI 0.121–0.790, *p* = 0.010). The model explained 96.1% of the variance in BFI at T6 (R-squared = 0.961).

Similarly, FACIT-F at T3 was strongly associated with FACIT-F at T6 (B = 1.138, 95% CI 1.028–1.248, *p* < 0.001; β = 0.914). In the conventional regression model, early GVHD was associated with a lower FACIT-F score at T6 (B = −1.763, 95% CI −3.376 to −0.149, *p* = 0.033). The model explained 98.1% of the variance in FACIT-F at T6 (R-squared = 0.981). Full estimates for the fatigue persistence models are presented in Table 4.

Because residual normality was less satisfactory in the fatigue models, heteroscedasticity-consistent HC3 standard errors were examined. The association between BFI at T3 and BFI at T6 remained robust, and the early GVHD effect also remained statistically significant using HC3 standard errors (*p* = 0.029). The association between FACIT-F at T3 and FACIT-F at T6 remained robust, whereas the GVHD association was attenuated and was no longer statistically significant (HC3 95% CI −4.148 to 0.622, *p* = 0.141). Detailed model diagnostics and HC3 robust-standard-error sensitivity analyses are provided in Supplementary Table S2. In additional leave-one-out sensitivity analyses, cross-validated Q^2^ was 0.9506 for the BFI T6 model (M4; RMSE = 0.34) and 0.9753 for the FACIT-F T6 model (M5; RMSE = 1.43). Although these findings indicated high within-cohort stability, the residual distributions of both fatigue models departed from normality, and the association between early GVHD and FACIT-F T6 was not statistically robust to HC3 standard errors (95% CI −4.148 to 0.622).

### 3.6. Early GVHD Treatment Response and 24-Month Outcomes

Among the 14 participants who developed early GVHD, 24-month PROs were descriptively better among treatment responders than among non-responders. At T6, mean FACT-BMT was 100.38 ± 22.66 among responders compared with 76.50 ± 22.95 among non-responders. The estimated difference between non-responders and responders was −23.88 points (95% CI −50.68 to 2.93, *p* = 0.076).

The corresponding mean BFI values were 5.66 ± 1.26 and 7.22 ± 0.23. Non-responders had a mean BFI score 1.55 points higher than responders (95% CI 0.41–2.70, *p* = 0.012). FACIT-F at T6 was also lower among non-responders (16.58 ± 3.65) than among responders (23.62 ± 6.31), corresponding to a mean difference of −7.04 points (95% CI −13.36 to −0.73, *p* = 0.032). Because of the small responder and non-responder subgroups, these estimates were interpreted as exploratory (Table 5). Because these estimates were based on only eight responders and six non-responders, the observed differences should be viewed as exploratory and hypothesis-generating. No multivariable responder-specific effect model was fitted.

### 3.7. Early Clinical Characteristics and 24-Month QoL

Longer hospitalization was associated with substantially lower 24-month FACT-BMT in unadjusted analysis. Mean FACT-BMT at T6 was 92.08 among participants with longer hospitalization and 130.75 among those with shorter hospitalization, corresponding to an unadjusted difference of −38.67 points (95% CI −60.02 to −17.31, *p* < 0.001).

However, after FACT-BMT at T3 and early GVHD were included in the model, hospitalization duration was no longer associated with FACT-BMT at T6 after adjustment for FACT-BMT at T3 and early GVHD (B = −1.366, 95% CI −4.803 to 2.072, *p* = 0.420). FACT-BMT at T3 and early GVHD remained associated with the outcome in the adjusted model.

Participants with documented weight loss had a lower mean FACT-BMT at T6 than those without documented weight loss (109.93 vs. 119.08), although the unadjusted between-group difference was not statistically significant (B = −9.14, 95% CI −35.12 to 16.83, *p* = 0.476). When documented weight loss was added to the primary multivariable model, it was associated with lower FACT-BMT at T6 (B = −3.604, 95% CI −5.536 to −1.673, *p* < 0.001), while FACT-BMT at T3 and early GVHD remained associated with the long-term outcome in the adjusted model. These clinical-variable models were considered exploratory and are summarized in Table 6.

### 3.8. Exploratory Analyses of Immediate Post-Transplant Status and Early Change

PROs obtained at T1 were also associated with 24-month QoL. Higher FACT-BMT at T1 was associated with higher FACT-BMT at T6 after adjustment for early GVHD (B = 0.858, 95% CI 0.602–1.114, *p* < 0.001). Higher BFI at T1 was associated with lower FACT-BMT at T6 (B = −11.418, 95% CI −19.438 to −3.398, *p* = 0.007), whereas higher FACIT-F at T1 was associated with higher FACT-BMT at T6 (B = 2.693, 95% CI 1.278–4.108, *p* < 0.001).

In contrast, the magnitude of change between T1 and T3 was not clearly associated with the corresponding 24-month outcomes after adjustment for early GVHD. Change in FACT-BMT from T1 to T3 was not significantly associated with FACT-BMT at T6 (B = 0.443, 95% CI −0.247 to 1.133, *p* = 0.198). Similarly, change in BFI was not associated with BFI at T6 (B = 0.253, 95% CI −0.528 to 1.035, *p* = 0.510), and change in FACIT-F was not associated with FACIT-F at T6 (B = 0.278, 95% CI −0.605 to 1.162, *p* = 0.523). Detailed estimates from the exploratory T1 and T1-to-T3 change-score analyses are presented in Supplementary Table S3.

### 3.9. Attrition and Sensitivity Analyses

Participants with available T6 outcomes were descriptively compared with the 13 participants without complete long-term follow-up. Among T6 completers, 17 of 28 participants (60.7%) were women compared with 7 of 13 (53.8%) among non-completers. The corresponding proportions in the younger age group were 46.4% and 38.5%.

Mean FACT-BMT at T1 was 79.4 ± 27.2 among T6 completers and 72.5 ± 15.4 among non-completers. Mean FACT-G was 46.4 ± 14.8 and 44.9 ± 10.0, respectively. Mean BFI was 6.9 ± 1.3 versus 7.1 ± 0.9, and FACIT-F was 18.14 ± 7.30 versus 17.7 ± 5.2. Early GVHD occurred in 50.0% of T6 completers and 53.8% of non-completers. Among the 13 participants without long-term follow-up, eight died before completion of follow-up and five were lost to follow-up, changed treatment center, or had no further contact. The descriptive comparison of T6 completers and non-completers is presented in Supplementary Table S4.

Model diagnostics for the primary analysis did not indicate major violations of linear regression assumptions. Residuals from the primary FACT-BMT model showed no clear departure from normality (Shapiro–Wilk *p* = 0.707) or evidence of heteroscedasticity (Breusch–Pagan *p* = 0.349). VIFs were approximately 1.94 in the primary model. The residuals-versus-fitted-values plot for the primary regression model is provided in Supplementary Figure S2.

Two observations exceeded the conventional Cook’s distance threshold of 4/N. Sensitivity analyses excluding either or both observations produced essentially unchanged estimates. After exclusion of both observations, FACT-BMT at T3 remained strongly associated with FACT-BMT at T6 (B = 1.078, *p* < 0.001), and early GVHD remained associated with lower FACT-BMT at T6 in the sensitivity analysis (B = −6.311, *p* < 0.001). HC3 robust-standard-error analyses were also materially consistent with the principal QoL models. Detailed regression diagnostics and HC3 sensitivity results are provided in Supplementary Table S2.

The original primary estimates were also reproduced by independent regression refitting. The primary model had variance inflation factors of 1.94 for each predictor, and the other four models had VIFs between 1.80 and 2.47. Two observations (P14 and P15) exceeded Cook’s distance 4/n in M1. Excluding both observations in a sensitivity-only analysis yielded B = 1.078 for FACT-BMT T3, B = −6.311 for early GVHD, and R^2^ = 0.9915 (n = 26), compared with B = 1.077, B = −6.133, and R^2^ = 0.9924 in the full n = 28 model. A separate summary of all LOOCV and bootstrap analyses is provided in Supplementary Table S1, with the agreement between observed and LOOCV-predicted 24-month FACT-BMT scores illustrated in Supplementary Figure S3; additional case-deletion estimates are presented in Supplementary Table S5.

## 4. Discussion

In this prospective longitudinal study, patient-reported health at 6 months after allogeneic hematopoietic stem cell transplantation (allo-HSCT) was strongly associated with quality of life and fatigue assessed 24 months after transplantation. Higher FACT-BMT at the 6-month assessment was associated with better long-term quality of life (QoL), and this association remained evident after accounting for early graft-versus-host disease (GVHD). Greater fatigue at 6 months, assessed using both the Brief Fatigue Inventory (BFI) and Functional Assessment of Chronic Illness Therapy—Fatigue (FACIT-F), was also associated with poorer 24-month QoL and with greater subsequent fatigue burden. Early GVHD remained associated with less favorable long-term outcomes in several models, whereas exploratory analyses of GVHD treatment response indicated poorer fatigue-related outcomes among non-responders. Taken together, these findings suggest that the 6-month post-transplant assessment may represent an informative clinical landmark for characterizing subsequent survivorship outcomes.

The 6-month post-transplant period may represent an important transition between acute recovery and longer-term survivorship. Huang et al. prospectively assessed older allo-HCT recipients before transplantation and at 3, 6, and 12 months and observed that mean functional status generally declined during the first 3–6 months, with recovery toward baseline by 12 months. In addition, early functional decline was associated with persistence of impairment at later assessments [17]. These findings support the clinical relevance of the 6-month assessment as a point at which recovery may still be incomplete and interindividual differences may remain substantial.

In the present cohort, QoL at 6 months was strongly associated with QoL at 24 months. This finding should not be interpreted as indicating that long-term outcome is predetermined at 6 months. Rather, patient-reported health at this stage may reflect the cumulative influence of treatment-related morbidity, physical functioning, symptom burden, GVHD-related limitations, and psychosocial adaptation. The persistence of the association after inclusion of early GVHD suggests that the patient’s own assessment of health at 6 months may contain clinically relevant information that is not fully captured by a binary classification of whether GVHD occurred during early follow-up.

Long-term QoL after transplantation is nevertheless dynamic. Ortolá-Alonso et al. followed patients undergoing hematopoietic stem cell transplantation with repeated FACT-BMT assessments for up to 24 months and documented substantial changes over time, together with considerable attrition; only 39 of 121 initially enrolled patients completed the 24-month follow-up [18]. Because that cohort included both autologous and allogeneic recipients, direct comparison with an allo-HSCT-only population should be made cautiously. Nonetheless, the study illustrates both the prolonged nature of post-transplant recovery and the methodological challenges inherent in long-term PRO follow-up.

The exceptionally high correlations and in-sample R^2^ values observed in the present study require particular caution. Re-analysis of the available participant-level data reproduced these estimates, and leave-one-out and case-deletion sensitivity analyses yielded similar within-cohort associations. Nevertheless, apparent model fit and internal stability do not demonstrate that a clinically useful prediction rule has been validated in new patients. The high concordance may partly reflect relatively stable between-patient ordering within a small, survivor-selected cohort, and it may not be replicated in independent datasets. Furthermore, unmeasured changes in clinical and psychosocial status between T3 and T6, including changes in GVHD activity, relapse, infections, immunosuppressive treatment, and functional status, remain potential sources of residual confounding. Future studies involving larger independent cohorts and more comprehensive longitudinal assessments are therefore needed to determine the reproducibility and clinical relevance of these associations.

Exploratory analyses also indicated that patient-reported status assessed immediately after transplantation was associated with 24-month QoL, whereas the magnitude of change between T1 and T3 was not clearly associated with the corresponding long-term outcomes after adjustment for early GVHD. These findings should be interpreted cautiously because T1 was obtained after conditioning and transplantation and therefore does not represent a true pre-transplant baseline. Moreover, change-score analyses may be influenced by measurement variability, baseline values, and regression to the mean. Accordingly, these findings should be considered exploratory and require confirmation in larger cohorts with true pre-transplant assessments.

Fatigue remains an important component of long-term morbidity after allo-HSCT and may reflect a combination of physiological, functional, and psychosocial burden. Dirou et al. evaluated relapse-free allo-HSCT survivors approximately one year after transplantation and demonstrated a high prevalence of impaired exercise capacity and physical deconditioning, supporting the close relationship between long-term symptom burden and functional recovery [19].

In the present study, greater fatigue at 6 months was associated with poorer QoL at 24 months. Higher BFI scores were associated with lower subsequent FACT-BMT, whereas higher FACIT-F scores, indicating a lower fatigue burden, were associated with better long-term QoL. The consistency of the overall direction across two complementary fatigue measures strengthens the interpretation that persistent fatigue during early survivorship may identify patients experiencing broader incomplete recovery rather than an isolated symptom.

Fatigue also demonstrated marked continuity between the 6- and 24-month assessments. Both BFI and FACIT-F measured at 6 months were strongly associated with their corresponding 24-month outcomes. This pattern supports continued symptom surveillance beyond the most intensive early post-transplant period, particularly among patients who continue to report substantial fatigue at approximately 6 months.

The BFI and FACIT-F-based models were not identical, and the clinical contribution of early GVHD differed across these analyses. Such differences should not necessarily be considered contradictory because the instruments capture related but not identical aspects of fatigue. The BFI emphasizes fatigue severity and interference with daily functioning, whereas FACIT-F reflects a broader patient-reported experience of fatigue and fatigue-related functioning. In a small cohort, differences in scale properties and the distribution of scores may also influence multivariable estimates.

GVHD is a major contributor to long-term symptom burden and impaired functioning after allo-HSCT. In a large cohort of transplant survivors, Lee et al. demonstrated clear differences in patient-reported health according to chronic GVHD status, with active chronic GVHD associated with a greater symptom burden and poorer functional outcomes [20]. Longitudinal data from the Chronic GVHD Consortium further indicate that changes in clinician- or NIH-assessed GVHD severity do not always parallel changes in patient-reported quality of life. Pidala et al. found that patient-reported changes in GVHD severity were more consistently related to changes in QoL than changes based on NIH global severity, supporting the concept that clinical disease assessment and PROs represent related but non-interchangeable dimensions of recovery [21].

In the primary model of the present study, early GVHD remained associated with lower 24-month FACT-BMT after accounting for FACT-BMT at 6 months. A similar association was observed in the BFI-based QoL model. These findings suggest that early GVHD may provide additional clinical information beyond the patient’s perceived health status at the 6-month landmark. At the same time, this association should be interpreted as conditional rather than causal, because the 6-month PRO itself may already reflect part of the burden resulting from early GVHD.

The relationship was not uniform across all models. After inclusion of FACIT-F, the association between early GVHD and 24-month FACT-BMT was attenuated and was no longer statistically significant. One possible interpretation is that part of the longer-term burden associated with GVHD is already expressed through fatigue-related functional impairment reported by patients at 6 months. This interpretation is consistent with previous evidence showing that clinician-rated GVHD and patient-reported health overlap but are not interchangeable [20,21].

The pooled indicator of early GVHD combined clinically distinct acute and chronic GVHD manifestations and did not account for phenotype-specific disease severity or changes in GVHD activity over time. Importantly, no active GVHD was documented at T6 (24 months post-transplantation) among the 28 participants included in the longitudinal analysis. Therefore, the observed associations with 24-month patient-reported outcomes should be interpreted in relation to documented GVHD history during early follow-up, rather than as evidence of concurrent active GVHD at T6. These findings should also be interpreted cautiously given the clinical heterogeneity of GVHD and the limited sample size.

Exploratory treatment-response analyses also suggested less favorable long-term outcomes among GVHD non-responders. Non-responders had poorer 24-month fatigue-related outcomes than responders, whereas the between-group difference in FACT-BMT was large in magnitude but estimated imprecisely. These results should be interpreted cautiously because only 14 participants developed GVHD and the responder and non-responder subgroups were small. In addition, treatment response was established during follow-up rather than representing a fixed baseline exposure. Accordingly, these analyses should be viewed as exploratory associations between clinical course and long-term patient-reported outcomes rather than as causal estimates of treatment-response effects.

From a clinical perspective, the present findings support the potential value of structured PRO assessment during early survivorship. Shaw et al. demonstrated that centralized repeated PRO collection after HCT is feasible, including assessments before transplantation, at day 100, and at 6 and 12 months, and that PROs can provide clinically meaningful information complementary to conventional clinical data [22].

The 6-month assessment may therefore represent an appropriate point for identifying patients who continue to experience substantial QoL impairment or fatigue and who may benefit from more detailed evaluation. Importantly, this should not be interpreted as assigning patients a fixed prognosis on the basis of a single questionnaire result. Rather, persistent impairment in PROs may serve as a signal prompting assessment of potentially modifiable contributors such as physical deconditioning, psychological distress, sleep disturbance, nutritional problems, ongoing immunosuppression, or GVHD-related functional limitations.

In clinical practice, persistently impaired PROs around six months could trigger a structured review of fatigue, physical functioning, sleep, psychological distress, and GVHD-related symptoms, followed by individualized rehabilitation or other supportive interventions as appropriate. Such an approach is proposed as a care pathway for further evaluation, not as a validated patient-level risk score or a threshold demonstrated by this small observational study.

Rehabilitation may form one component of such individualized supportive care. A recent allo-HSCT-specific systematic review concluded that physical therapy and exercise interventions across the transplant trajectory are generally feasible and safe, although the certainty and consistency of benefits differ by outcome and treatment phase [23]. A broader systematic review and meta-analysis of exercise following HSCT found more consistent benefits for functional capacity and lower-limb strength than for fatigue and quality of life [24]. Therefore, identification of impaired QoL or persistent fatigue should prompt individualized assessment and targeted supportive care rather than imply that any single intervention will necessarily normalize long-term patient-reported outcomes.

Long-term PRO research after transplantation is particularly vulnerable to attrition. In the longitudinal study by Ortolá-Alonso et al., participation decreased from 121 patients at enrollment to 39 participants at 24 months [18]. Even in larger registry-based initiatives, repeated PRO completion is incomplete; Shaw et al. reported that centralized longitudinal PRO collection was feasible but response rates varied across follow-up assessments [22].

This issue is directly relevant to the present study. Of the 41 participants enrolled in the parent cohort, 28 contributed data to the 24-month analytical sample. Eight patients died before completion of follow-up, while five were lost to follow-up, changed treatment center, or had no further contact. Consequently, the present findings describe prospective associations among survivors with available long-term PRO data rather than the experience of the complete original transplant population.

Attrition should not be treated as a single homogeneous form of missing data. The eight deaths before the long-term assessment represent a distinct process from the five participants who did not complete follow-up for non-death reasons. Death makes the planned 24-month patient-reported outcome undefined rather than merely unrecorded. Furthermore, participants who survived and completed the T6 assessment may differ systematically from those who died or disengaged from follow-up. Available T1 measurements showed a modestly lower mean FACT-BMT score (72.50 vs. 79.43) and a slightly higher mean BFI score (7.10 vs. 6.86) among participants without T6 data compared with those who completed follow-up. However, these descriptive comparisons cannot exclude informative attrition or survivorship-related selection. Consequently, the observed associations should be interpreted as conditional on survival and completion of the 24-month assessment and should not be assumed to represent the entire originally enrolled transplant population.

The present 24-month findings can also be considered alongside our previously published early-phase analysis of the same prospective cohort. In that report, Lampart et al. documented improving quality of life and decreasing fatigue during the first 6 months after allo-HSCT, together with consistently less favorable patient-reported outcomes among participants who developed GVHD [25]. Because the two analyses involve overlapping participants, the earlier publication provides context for the study trajectory rather than independent replication of the associations between 6- and 24-month outcomes reported here. Independent cohorts further highlight the importance of repeated assessments during recovery: Pawełczak-Szastok et al. observed a pronounced decline in FACT-BMT scores during transplantation-related hospitalization, with only partial recovery by discharge in a cohort comprising both autologous and allogeneic recipients [26]. In 189 allo-HSCT recipients, Eriksson et al. reported that a higher symptom burden at 4 months was associated with poorer general health at 1 year [27]. Although the timing and outcomes differed across studies, these findings provide a clinical rationale for reassessing patient-reported status at approximately 6 months without implying that an individual long-term prognosis can be established from a single assessment.

The relevance of persistent symptoms also extends beyond overall quality-of-life scores. In the randomized BMT CTN 1703 study, Holtan et al. reported a lower patient-reported chronic GVHD symptom burden during the first post-transplant year with post-transplant cyclophosphamide-based prophylaxis than with tacrolimus/methotrexate, although the assessed PROMIS domains did not show significant between-group differences [28]. Chiang et al., in a cohort including autologous and allogeneic HSCT recipients, found that greater fatigue was associated with delayed return to work, while joint/fascia chronic GVHD was associated with a lower likelihood of work resumption within the allogeneic subgroup [29]. Furthermore, a recent nationwide Danish survey of 895 allo-HCT survivors documented substantial long-term symptom burden, with fatigue reported by 90% of respondents [30]. Together, these observations emphasize that survival and resolution of active GVHD do not necessarily imply full functional recovery. They support linking repeated PRO assessment to clinical review and individualized supportive care, while neither these external findings nor our observational data establish that interventions triggered at 6 months will improve 24-month outcomes.

The present study has several strengths. First, it is based on a prospective longitudinal cohort with predefined patient-reported outcome assessments extending to 24 months after allo-HSCT. Second, QoL and fatigue were evaluated using validated and complementary instruments, allowing assessment of both transplant-specific quality of life and fatigue burden. Third, patient-reported measures were integrated with clinically characterized GVHD and treatment-response data, allowing patient-reported and clinical indicators of long-term outcome to be evaluated within the same analytical framework. Fourth, the 6-month assessment was selected as the primary early landmark, representing a clinically relevant stage between the intensive early post-transplant period and longer-term survivorship. This allowed the study to address a question distinct from conventional pre-transplant prognostic analyses and from analyses focused primarily on longitudinal trajectories. Although the models were intentionally restricted to two prespecified explanatory variables, parsimonious specification does not overcome the limited precision of a small cohort, residual confounding, or survivor selection. Internal resampling estimates were relatively stable, but this does not substitute for independent external validation. Findings from GVHD-treatment responder/non-responder comparisons are particularly uncertain because of the group sizes (n = 8 and n = 6).

Several limitations should also be considered. First, the relatively small single-center cohort limits statistical power, the precision of effect estimates, and generalizability. This limitation is particularly relevant to analyses of GVHD treatment response, in which the number of patients within individual subgroups was small and confidence intervals were consequently wide. Multivariable models were therefore intentionally restricted to a small number of clinically justified variables.

Second, the 24-month analysis is inherently susceptible to attrition and survivor bias. Of the 41 participants enrolled in the parent cohort, long-term PRO data were available for 28 participants, whereas eight patients died before completion of follow-up and five were lost to follow-up, changed treatment center, or had no further contact. Patients contributing 24-month PRO data therefore represent a selected subset of the original transplant population. Although early demographic, clinical, and patient-reported characteristics were descriptively compared between participants with and without available long-term follow-up, informative missingness cannot be excluded. In particular, death before the 24-month assessment represents structural unavailability of PRO data rather than conventional questionnaire missingness, and no hypothetical long-term PRO values were assigned to deceased participants.

Third, the first PRO assessment was performed within 24 h after transplantation, after conditioning therapy, and therefore does not represent a true pre-transplant baseline. Analyses involving T1 and changes between T1 and T3 should consequently be interpreted as exploratory assessments of immediate post-transplant status and early recovery rather than as changes from a pre-transplant baseline.

Fourth, the limited sample size restricted the number of potential confounders that could be included simultaneously. Factors occurring between the 6- and 24-month assessments, including changes in GVHD activity, immunosuppressive treatment, comorbidity, infections, functional status, and other post-transplant complications, may influence long-term patient-reported outcomes and were not fully captured in the present models. Residual confounding therefore remains possible.

Fifth, although regression diagnostics and sensitivity analyses supported the overall robustness of the principal associations, the observational design precludes causal inference. Associations between early PROs, GVHD characteristics, and 24-month outcomes should therefore not be interpreted as evidence that the early factors directly caused subsequent differences in QoL or fatigue.

Finally, the present study was designed to examine prospective associations rather than to develop or validate a clinical prediction model. Accordingly, the findings should be interpreted as evidence that selected early patient-reported and clinical characteristics may be informative regarding longer-term outcomes, rather than as a validated tool for individual risk prediction.

## 5. Conclusions

Among 28 patients with available 6- and 24-month patient-reported outcomes after allo-HSCT, QoL and fatigue measured at six months were strongly associated with their corresponding 24-month outcomes. Early GVHD history was associated with less favorable long-term QoL in selected models, whereas differences by GVHD treatment response were exploratory and imprecisely estimated. Although the unusually high correlations were reproduced and showed internal stability in sensitivity analyses, these findings do not establish an externally validated prediction model and may not generalize beyond survivors who completed long-term follow-up. A six-month PRO assessment may be a useful prompt for clinical review and individualized supportive care, but this potential application requires confirmation in larger independent cohorts.

## Figures and Tables

**Table 1:** Characteristics of the 24-month analytical cohort (n = 28).

Variable	Category	n	%
Sex	Female	17	60.7
	Male	11	39.3
Age group	Younger	13	46.4
	Older	15	53.6
Diagnosis	AML	16	57.1
	CML	7	25.0
	ALL	4	14.3
	MDS	1	3.6
Documented weight loss	Yes	15	53.6
	No	13	46.4
Hospitalization duration	Shorter (<42 days)	16	57.1
	Longer (≥42 days)	12	42.9
Early GVHD	No	14	50.0
	Yes	14	50.0
GVHD response (n = 14)	Responder	8	57.1
	Non-responder	6	42.9

AML, acute myeloid leukemia; CML, chronic myeloid leukemia; ALL, acute lymphoblastic leukemia; MDS, myelodysplastic syndrome; GVHD, graft-versus-host disease. Younger age group: <48 years; older age group: ≥48 years. Shorter hospitalization: <42 days; longer hospitalization: ≥42 days.

**Table 2:** Patient-reported outcomes at 6 months (T3) and 24 months (T6), overall and by early GVHD/response group.

Group	Outcome	Timepoint	n	Mean	SD
Overall	FACT-BMT	T3	28	104.11	28.47
Overall	FACT-BMT	T6	28	114.18	33.05
Overall	BFI	T3	28	5.50	1.20
Overall	BFI	T6	28	5.20	1.57
Overall	FACIT-F	T3	28	24.79	7.45
Overall	FACIT-F	T6	28	27.91	9.28
No GVHD	FACT-BMT	T3	14	123.57	19.33
No GVHD	FACT-BMT	T6	14	138.21	19.87
GVHD responder	FACT-BMT	T3	8	92.38	20.72
GVHD responder	FACT-BMT	T6	8	100.38	22.66
GVHD non-responder	FACT-BMT	T3	6	74.33	21.48
GVHD non-responder	FACT-BMT	T6	6	76.50	22.95
No GVHD	BFI	T3	14	4.71	0.83
No GVHD	BFI	T6	14	4.08	0.93
GVHD responder	BFI	T3	8	5.75	1.04
GVHD responder	BFI	T6	8	5.66	1.26
GVHD non-responder	BFI	T3	6	7.00	0.00
GVHD non-responder	BFI	T6	6	7.22	0.23
No GVHD	FACIT-F	T3	14	30.43	4.31
No GVHD	FACIT-F	T6	14	35.21	4.92
GVHD responder	FACIT-F	T3	8	21.25	5.85
GVHD responder	FACIT-F	T6	8	23.62	6.31
GVHD non-responder	FACIT-F	T3	6	16.33	2.94
GVHD non-responder	FACIT-F	T6	6	16.58	3.65

Higher FACT-BMT and FACIT-F scores indicate better HRQoL and lower fatigue burden, respectively; higher BFI scores indicate greater fatigue. The zero standard deviation for T3 BFI in non-responders reflects six identical recorded scores of 7.0 (n = 6).

**Table 3:** Univariable associations between 6-month PROs and 24-month FACT-BMT.

Predictor	B	95% CI Low	95% CI High	*p*-Value	R-Squared
FACT-BMT T3	1.154	1.102	1.205	<0.001	0.988
BFI T3	−21.936	−28.622	−15.250	<0.001	0.636
FACIT-F T3	3.861	2.980	4.742	<0.001	0.757

**Table 4:** Prespecified multivariable models for 24-month QoL and fatigue outcomes.

Model/Outcome	Predictor	B	95% CI Low	95% CI High	*p*-Value	Standardized β	R-Squared
M1: FACT-BMT T6	FACT-BMT T3	1.077	1.019	1.135	<0.001	0.928	0.992
M1: FACT-BMT T6	Early GVHD	−6.133	−9.373	−2.893	<0.001	−0.094	0.992
M2: FACT-BMT T6	BFI T3	−15.046	−23.149	−6.943	<0.001	−0.547	0.715
M2: FACT-BMT T6	Early GVHD	−24.428	−43.553	−5.302	0.014	−0.376	0.715
M3: FACT-BMT T6	FACIT-F T3	3.275	1.896	4.654	<0.001	0.738	0.769
M3: FACT-BMT T6	Early GVHD	−11.110	−31.289	9.069	0.268	−0.171	0.769
M4: BFI T6	BFI T3	1.142	1.000	1.284	<0.001	0.876	0.961
M4: BFI T6	Early GVHD	0.455	0.121	0.790	0.010	0.148	0.961
M5: FACIT-F T6	FACIT-F T3	1.138	1.028	1.248	<0.001	0.914	0.981
M5: FACIT-F T6	Early GVHD	−1.763	−3.376	−0.149	0.033	−0.097	0.981

All models included n = 28. M1 was the prespecified primary model. BFI and FACIT-F were modeled separately. Conventional OLS *p*-values are shown; HC3 robust-standard-error sensitivity results are provided in Supplementary Table S2.

**Table 5:** Twenty-four-month outcomes by early GVHD treatment response (n = 14).

Outcome	Responder (n = 8)	Non-Responder (n = 6)	Difference (NR − R)	95% CI	*p*-Value
FACT-BMT T6	100.38 ± 22.66	76.50 ± 22.95	−23.88	−50.68 to 2.93	0.076
BFI T6	5.66 ± 1.26	7.22 ± 0.23	1.55	0.41 to 2.70	0.012
FACIT-F T6	23.62 ± 6.31	16.58 ± 3.65	−7.04	−13.36 to −0.73	0.032

Response analysis was exploratory because of the small subgroup sizes. Positive BFI differences indicate greater fatigue in non-responders.

**Table 6:** Clinical sensitivity analyses for FACT-BMT at 24 months.

Model	Predictor	B	95% CI Low	95% CI High	*p*-Value
Primary + hospitalization	FACT-BMT T3	1.053	0.970	1.137	<0.001
Primary + hospitalization	Early GVHD	−6.868	−10.624	−3.112	<0.001
Primary + hospitalization	Longer hospitalization (≥42 days)	−1.366	−4.803	2.072	0.420
Primary + documented weight loss	FACT-BMT T3	1.087	1.040	1.134	<0.001
Primary + documented weight loss	Early GVHD	−4.990	−7.667	−2.314	<0.001
Primary + documented weight loss	Documented weight loss	−3.604	−5.536	−1.673	<0.001

Clinical-variable models were prespecified as exploratory/sensitivity analyses to preserve model parsimony.

## Data Availability

The data supporting the findings of this study are available from the authors upon request.

## Supplementary Materials

The following supporting information can be downloaded at: https://www.mdpi.com/article/10.3390/cancers18213542/s1, Table S1. Independent model reproduction, LOOCV performance, VIF, and bootstrap stability across five regression models. Table S2. Model diagnostics and HC3 robust-standard-error sensitivity analysis. Table S3. Exploratory associations of immediate post-transplant PROs and early PRO changes (T3 minus T1) with 24-month outcomes. Table S4. Descriptive comparison of T6 completers and non-completers using available early-phase data. Table S5. Influential-observation sensitivity analysis for the primary model. Figure S1. Participant-level 6- and 24-month FACT-BMT scores by early GVHD status. Figure S2. Primary-model residuals versus fitted outcomes. Figure S3. Primary model: observed outcomes versus leave-one-out predictions.

## Abbreviations

The following abbreviations are used in this manuscript:

allo-HSCT	Allogeneic hematopoietic stem cell transplantation
GVHD	Graft-versus-host disease
eGVHD	eGVHD application
PRO/PROs	Patient-reported outcome/patient-reported outcomes
QoL	Quality of life
FACT-BMT	Functional Assessment of Cancer Therapy—Bone Marrow Transplant
FACT-G	Functional Assessment of Cancer Therapy—General
FACIT-F	Functional Assessment of Chronic Illness Therapy—Fatigue
BFI	Brief Fatigue Inventory
AML	Acute myeloid leukemia
CML	Chronic myeloid leukemia
ALL	Acute lymphoblastic leukemia
MDS	Myelodysplastic syndrome
NIH	National Institutes of Health
CI/CIs	Confidence interval/confidence intervals
IQR/IQRs	Interquartile range/interquartile ranges
SD/SDs	standard deviation/standard deviations
VIF/VIFs	variance inflation factor/variance inflation factors
HC3	Heteroscedasticity-consistent type 3 robust standard errors
OLS	Ordinary least squares
ROC	Receiver operating characteristic
BP	Breusch–Pagan
Q–Q	Quantile–quantile (plot)
SPSS	Statistical Package for the Social Sciences
T1	Assessment within 24 h after allo-HSCT
T2	1-month assessment
T3	6-month assessment
T4	12-month assessment
T5	18-month assessment
T6	24-month assessment
R	Responder
NR	Non-responder
M1–M5	Model 1–Model 5
ΔPRO	change in patient-reported outcome, calculated as PRO T3 − PRO T1
