# Supplementary material for: Early Patient-Reported Outcomes and Graft-Versus-Host Disease as Indicators of Long-Term Quality of Life and Fatigue After Allogeneic Hematopoietic Stem Cell Transplantation: A 24-Month Prospective Study
Source: Cancers (Basel). 2026 Nov 4;18(21):3542. doi: 10.3390/cancers18213542 (PMC13762265; doi:10.3390/cancers18213542)
Supplement: Supplementary file 1 [file cancers-18-03542-s001.zip › cancers-4637373-supplementary.pdf]

Supplementary Table S1. Independent model reproduction, LOOCV performance, VIF, and bootstrap stability across five regression models.

Model	R^2	LOOCV Q^2	LOOCV RMSE	VIF	Bootstrap 95% CI: early PRO predictor	Bootstrap 95% CI: GVHD
M1	0.992	0.991	3.16	1.94	1.014 to 1.127	-9.051 to -3.198
M2	0.715	0.648	19.27	1.80	-21.720 to -7.323	-42.193 to -9.287
M3	0.769	0.712	17.41	2.47	1.813 to 4.439	-29.724 to 6.342
M4	0.961	0.951	0.34	1.80	0.989 to 1.295	0.080 to 0.815
M5	0.981	0.975	1.43	2.47	1.038 to 1.281	-3.318 to 0.368

M1: FACT-BMT T6 ~ FACT-BMT T3 + early GVHD; M2: FACT-BMT T6 ~ BFI T3 + early GVHD; M3: FACT-BMT T6 ~ FACIT-F T3 + early GVHD; M4: BFI T6 ~ BFI T3 + early GVHD; M5: FACIT-F T6 ~ FACIT-F T3 + early GVHD. All models n = 28. Q^2 is defined as 1 − PRESS/TSS, with each prediction obtained from leave-one-out refitting. Bootstrap intervals are nonparametric 2.5th–97.5th percentiles across 2,000 participant-level resamples. Each listed VIF applies to both model predictors and the intercept was excluded from the VIF range. Resampling stability does not constitute external validation.

Supplementary Table S2. Model diagnostics and HC3 robust-standard-error sensitivity analysis.

Model	Outcome	Shapiro p	BP p	Max Cook D	Cook > 4/N	Predictor	B	HC3 95% CI	HC3 p
M1 primary	FACT-BMT T6	0.707	0.349	0.199	P14, P15	FACT-BMT T3	1.077	1.016 to 1.138	<0.001
M1 primary	FACT-BMT T6	0.707	0.349	0.199	P14, P15	Early GVHD	-6.133	-9.382 to -2.884	<0.001
M2	FACT-BMT T6	0.074	0.486	0.216	P15	BFI T3	-15.046	-22.919 to -7.173	<0.001
M2	FACT-BMT T6	0.074	0.486	0.216	P15	Early GVHD	-24.428	-41.996 to -6.859	0.008
M3	FACT-BMT T6	0.202	0.061	0.252	P15	FACIT-F T3	3.275	1.858 to 4.692	<0.001
M3	FACT-BMT T6	0.202	0.061	0.252	P15	Early GVHD	-11.110	-29.686 to 7.466	0.229
M4	BFI T6	0.003	0.415	0.368	P17, P28	BFI T3	1.142	0.975 to 1.310	<0.001
M4	BFI T6	0.003	0.415	0.368	P17, P28	Early GVHD	0.455	0.050 to 0.860	0.029
M5	FACIT-F T6	0.026	0.542	0.463	P17, P20, P24	FACIT-F T3	1.138	0.980 to 1.296	<0.001
M5	FACIT-F T6	0.026	0.542	0.463	P17, P20, P24	Early GVHD	-1.763	-4.148 to 0.622	0.141

Supplementary Table S3. Exploratory associations of immediate post-transplant PROs and early PRO changes (T3 minus T1) with 24-month outcomes.

Outcome	Predictor	B	95% CI low	95% CI high	p-value	R-squared
FACT-BMT T6	FACT-BMT T1	0.858	0.602	1.114	<0.001	0.845
FACT-BMT T6	Early GVHD	-18.898	-32.551	-5.245	0.009	0.845
FACT-BMT T6	BFI T1	-11.418	-19.438	-3.398	0.007	0.664
FACT-BMT T6	Early GVHD	-30.129	-50.104	-10.153	0.005	0.664
FACT-BMT T6	FACIT-F T1	2.693	1.278	4.108	<0.001	0.720
FACT-BMT T6	Early GVHD	-20.370	-40.664	-0.077	0.049	0.720
FACT-BMT T6	Delta FACT-BMT T1-T3	0.443	-0.247	1.133	0.198	0.578
FACT-BMT T6	Early GVHD	-45.889	-63.589	-28.189	<0.001	0.578
BFI T6	Delta BFI T1-T3	0.253	-0.528	1.035	0.510	0.542
BFI T6	Early GVHD	2.250	1.392	3.108	<0.001	0.542
FACIT-F T6	Delta FACIT-F T1-T3	0.278	-0.605	1.162	0.523	0.649
FACIT-F T6	Early GVHD	-14.329	-18.864	-9.794	<0.001	0.649

Delta was calculated as T3 minus T1. These analyses were exploratory and were not used to define individual recovery categories or phenotypes.

Supplementary Table S4. Descriptive comparison of T6 completers and non-completers using available early-phase data.

Variable	T6 completers (n = 28)	T6 non-completers (n = 13)
Female sex, n (%)	17 (60.7)	7 (53.8)
Male sex, n (%)	11 (39.3)	6 (46.2)
Younger age group, n (%)	13 (46.4)	5 (38.5)
Older age group, n (%)	15 (53.6)	8 (61.5)
FACT-BMT T1, mean ± SD	79.4 ± 27.2	72.5 ± 15.4
FACT-G T1, mean ± SD	46.4 ± 14.8	44.9 ± 10.0
BFI T1, mean ± SD	6.9 ± 1.3	7.1 ± 0.9
FACIT-F T1, mean ± SD	18.14 ± 7.30	17.7 ± 5.2
Early GVHD, n (%)	14 (50.0)	7 (53.8)
Death before T6, n (%)	0	8 (61.5)
Loss/change of center/no further contact, n (%)	0	5 (38.5)

This comparison was descriptive and was intended to characterize possible attrition and survivor bias rather than to test formal hypotheses.

Supplementary Table S5. Influential-observation sensitivity analysis for the primary model.

Analysis	n	B(FACT-BMT T3)	B(early GVHD)	R^2
All participants	28	1.077	−6.133	0.9924
Exclude P14	27	1.092	−6.028	0.9931
Exclude P15	27	1.061	−6.493	0.9908
Exclude P14 and P15	26	1.078	−6.311	0.9915

P14 and P15 were flagged by Cook’s D > 4/n in the full primary model. The row exclusions were performed only as sensitivity analyses; all 28 participants remained in the prespecified primary anal

Supplementary Figure S1. Participant-level 6- and 24-month FACT-BMT scores by early GVHD status.

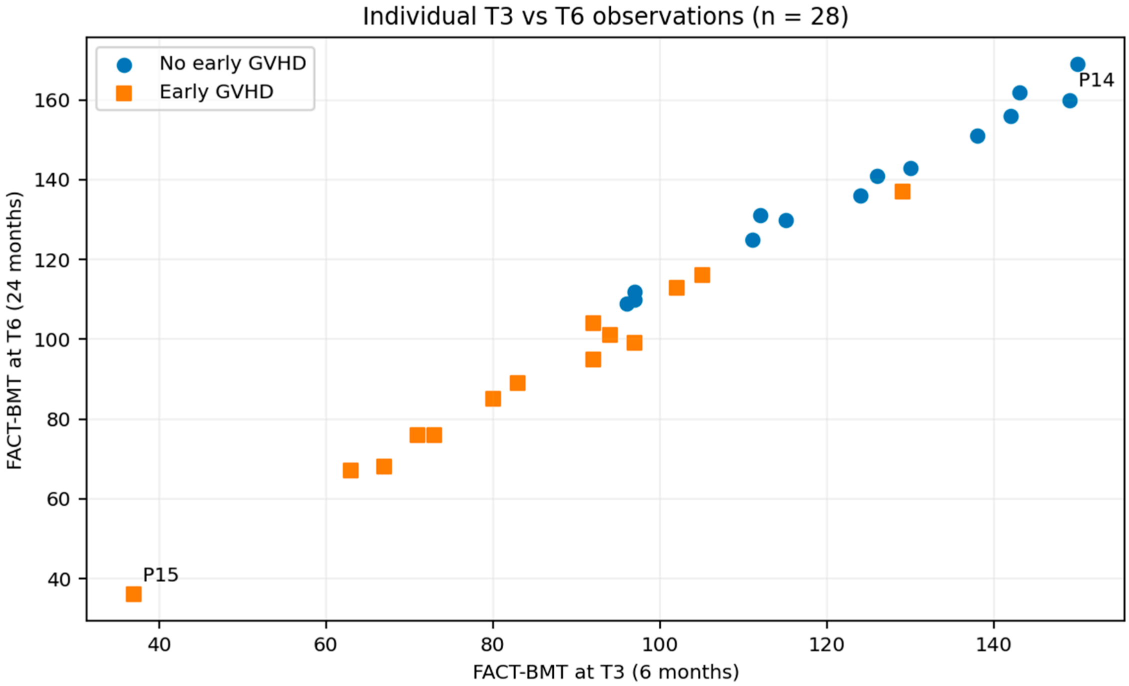

Individual paired T3 and T6 FACT-BMT scores, categorized by presence of early GVHD. Each plotted point represents one participant (n = 28). P14 and P15 are labeled because they exceeded Cook's D > 4/n in the primary adjusted regression.

Supplementary Figure S2. Primary-model residuals versus fitted outcomes.

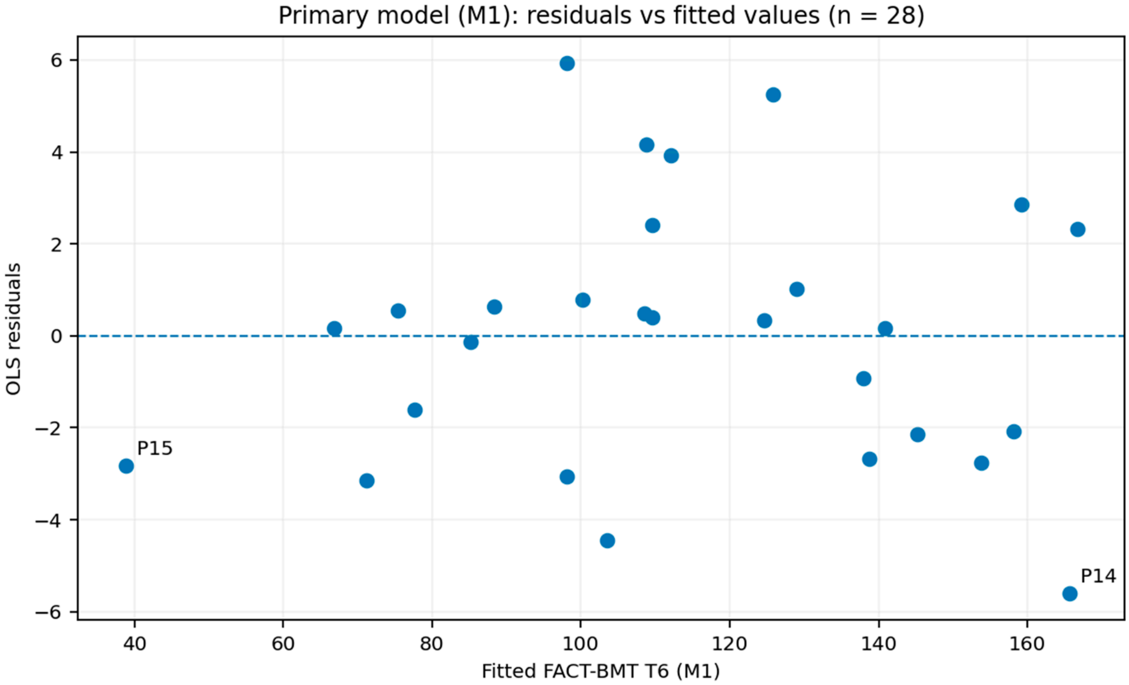

Residuals plotted against fitted FACT-BMT T6 for the adjusted primary model (M1; n = 28). Dashed line denotes zero residual.

Supplementary Figure S3. Primary model: observed outcomes versus leave-one-out predictions.

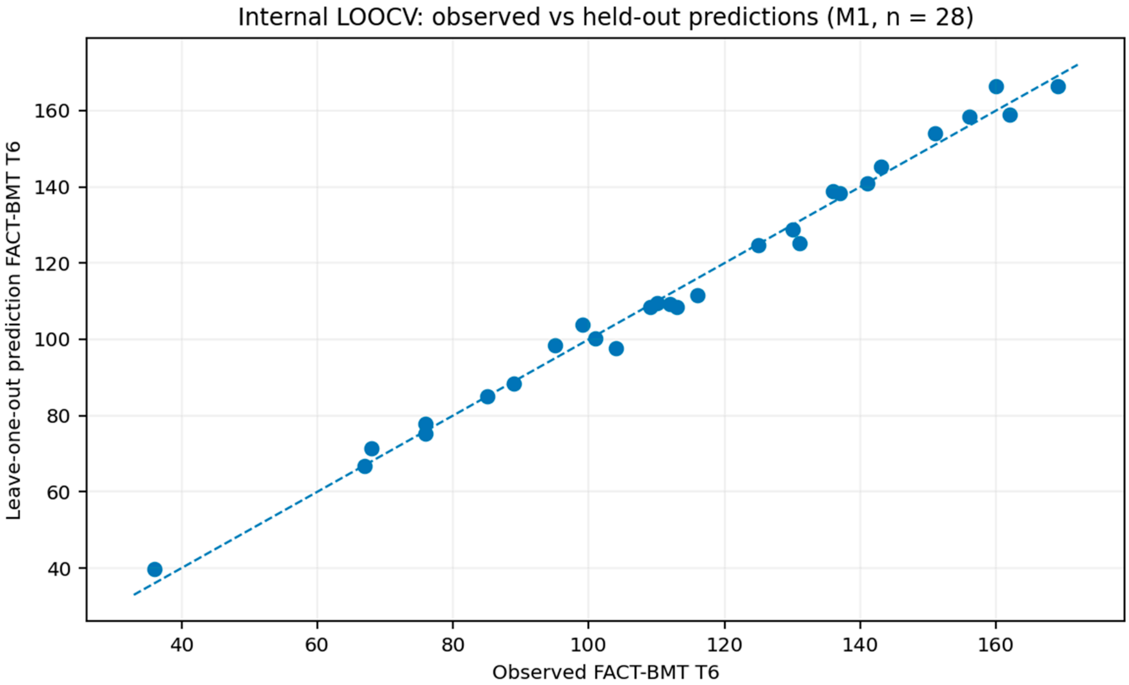

Observed FACT-BMT T6 scores versus predictions generated when the participant was excluded from model fitting (leave-one-out cross-validation; n = 28). Dashed line denotes perfect agreement.
